# A Rare Case of Rectus Muscle Twitching Due to Abandoned Uncapped Pacemaker Leads

**DOI:** 10.7759/cureus.49668

**Published:** 2023-11-29

**Authors:** Issa Al-Khdour, Amro AlAqra, Moath Nairat, Ibrahim Marai, Nadine Yaghi, Fateh Awwad

**Affiliations:** 1 Cardiac Surgery, An-Najah National University Hospital, Nablus, PSE; 2 Cardiology, An-Najah National University Hospital, Nablus, PSE; 3 General Surgery, Faculty of Medicine, Al-Quds University, Jerusalem, PSE; 4 Nursing, An-Najah National University Hospital, Nablus, PSE

**Keywords:** rectus, twiddler's syndrome, pacing wires, minimally invasive cardiac surgery, complications to pacemaker implantation

## Abstract

Extra-cardiac stimulation after cardiac pacemaker implantation is seldom seen in the cardiac field. However, this case report demonstrates an unusual symptom of persistent abdominal twitching in a 42-year-old male patient who underwent pacemaker replacement, lasting for 15 years. Initially, it was attributed to diaphragmatic pacing by the new pacemaker. Despite several attempts to replace the endocardial leads, the patient's symptoms did not improve. Finally, he was referred to our hospital, where our team conducted further investigations and discovered that the old pacemaker lead was exposed, leading to excitation of the rectus muscle.

## Introduction

The use of pacemakers in the management of various cardiac conditions is a common procedure that is used worldwide. However, despite being considered a routine procedure with well-known complications, rare complications such as stimulation of extra-cardiac structures might occur [1,2]. We present a unique case of an abandoned non-capped epicardial pacemaker lead that was initially connected to an abdominally placed generator in a 42-year-old male patient with a complex cardiac background from a young age. After the reimplantation of a new endocardial pacemaker system along with a new generator in the left shoulder, the patient started to experience persistent contractions of the rectus muscle. To the best of our knowledge, this is the first reported case of its kind (according to a PubMed search).

## Case presentation

A 42-year-old male patient with a complex cardiac background presented to the cardiac department of our hospital with a chief complaint of chronic spontaneous abdominal contractions of 15 years duration. Our patient’s cardiac history dates back to the age of 15, when he first underwent ventricular septal defect closure surgery that warranted the implantation of permanent epicardial leads with an abdominally attached generator due to the complication of a complete heart block. In 2007, when the patient turned 27 years old, he had a new dual chamber pacemaker implantation with an endocardial lead system by Medtronic (Medtronic Inc., Minneapolis, MN, USA), and a generator was positioned in the left shoulder area. Shortly after this procedure, he started to complain of continuous abdominal twitching. This led the patient to seek medical advice and diaphragmatic pacing from the new endocardial lead system was speculated to be the cause of his symptoms. Despite many trials of repositioning the endocardial leads, the patient’s symptoms endured without any improvement, and the abdominal pulsations did not terminate.

After numerous failed trials of treatment that included repositioning of the ventricular lead, the patient, now 42 years of age, was referred to the cardiac surgery department of our hospital for a thorough evaluation. Upon initial clinical examination, the presence of persistent, physically unprovoked abdominal pulsations was noted. No other signs or symptoms were reported. A differential diagnosis of diaphragmatic pacing, twiddler syndrome, or current leak was considered as the potential etiologies of our patient’s complaint.

Based on a consultation with the electrophysiology team, it was recommended to conduct an electrophysiology study. Thus, a temporary pacemaker electrode was inserted via the right femoral vein and introduced to the right ventricle. The electrode was localized at the high right ventricular outflow tract, mid right ventricular outflow tract, high septum, mid septum, and low septum. While pacing at the above-mentioned sites at different amplitudes, the abdominal contractions continued nonetheless. The frequency of abdominal contractions significantly decreased when the rate was reduced to 30 beats per minute. We could not, however, reprogram to pacing off mode because of a total pacing dependency.

After a multidisciplinary discussion between both the electrophysiology and cardiac surgery teams, it was concluded that the patient’s symptoms were due to the abandoned epicardial pacemaker wires, not the endocardial lead as previously anticipated. Our multidisciplinary team concluded that surgical extraction versus isolation of the abdominal part of the abandoned epicardial lead was the appropriate therapeutic intervention. A redo sternotomy with complete extraction of the wire was also considered a second option of management if the former attempt failed. The plan of management was thoroughly explained to the patient, who gave his full consent.

**Figure 1:**
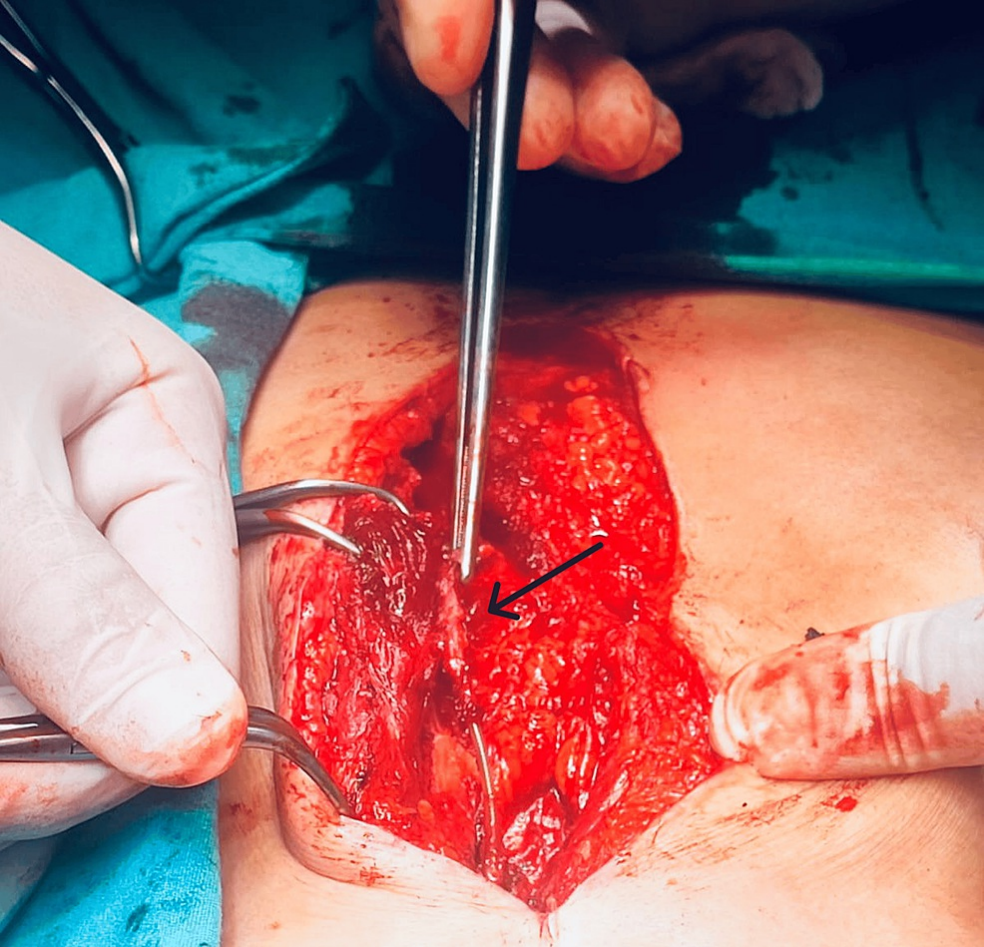
Intraoperative view of the old epicardial pacemaker lead (black arrow)

**Figure 2:**
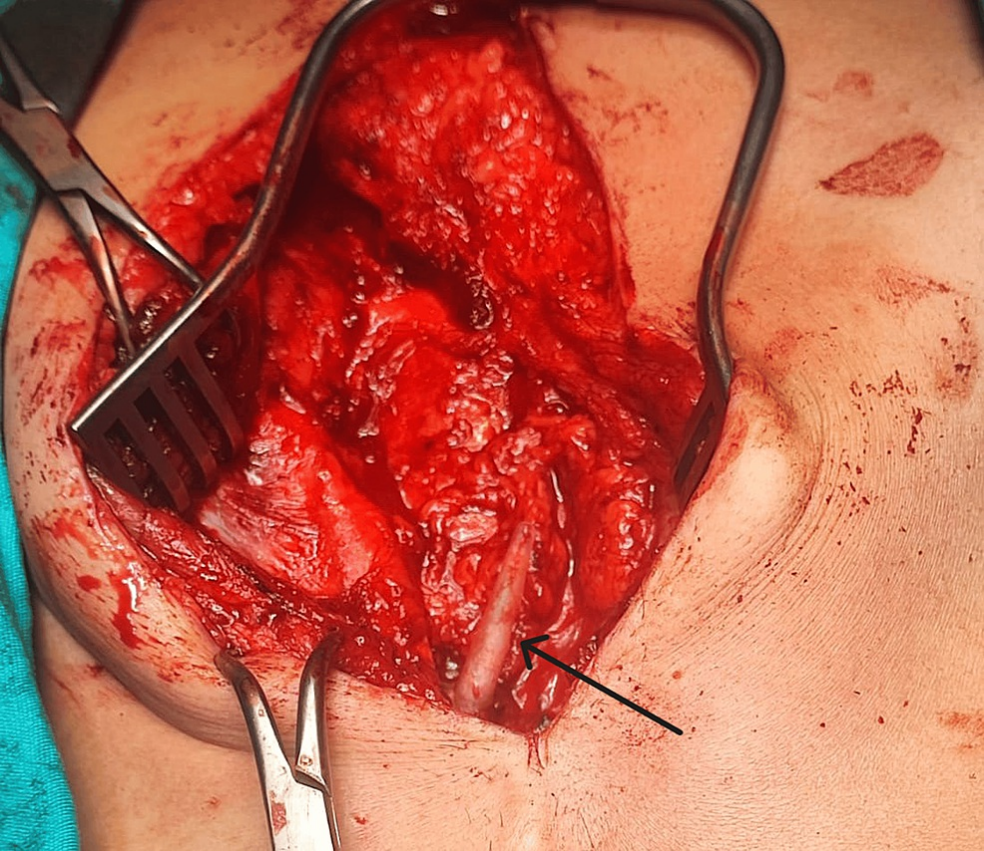
Intraoperative view of the old pacemaker lead after resection of its exposed part and isolating it with a lead cap (black arrow)

A successful isolation of the uncapped part of the pacemaker lead by a minimal surgical incision was performed, resulting in a complete recovery. The procedure involved a sub-xiphoid surgical incision, extending down to the upper abdomen, followed by careful dissection through the subcutaneous tissue in an attempt to localize the old lead. During the surgery, it was discovered that the pacemaker lead was severely adherent to the inferior surface of the upper part of the left rectus muscle (Figure 1). The lead was released from the muscle at about 10 cm, and after trimming the abdominal part of the lead, the tip of the residual lead was covered with a lead cap (Figure 2).

The surgery was uneventful, and the patient’s condition was fully monitored for two postoperative days, after which complete remission was confirmed by our team. The abdominal pulsations disappeared entirely after more than a decade of suffering, and the patient was discharged home in excellent medical condition.

## Discussion

Stimulation of extra-cardiac structures following pacemaker implantation is a rare occurrence that has been reported previously and is generally regarded as an uncommon complication. The published literature on this matter lists several possible complications, including the development of twiddler syndrome, the occurrence of a current leak, and phrenic nerve excitation causing diaphragmatic twitching [3-5]. 

In this case report, we describe an unusual incidence of persistent abdominal pulsations in an adult male patient, on the background of a misdiagnosis of diaphragmatic stimulation. After extensive evaluation that entailed a multidisciplinary approach and an electrophysiology study, the source of the patient’s main complaint was identified to be the old exposed epicardial pacemaker lead, which was consequently stimulating and inducing the rectus muscle to contract with each pacing simultaneously.

After careful consideration, we opted for surgical isolation and covering of the exposed part of the old wire, which not only resulted in the complete remission of 15 years of symptoms but also enriched our understanding of this rare electrophysiological complication. Despite initially debating a redo sternotomy, this simple salvage and cover technique was preferred over an invasive procedure, proved to be highly effective, and resulted in the total amelioration of the patient's condition. A similar case has been reported by Bohm et al. where muscle stimulation occurred in the left lower rib region after the implantation of an endocardial system; it was addressed by cutting and capping the old epicardial lead. In their case, the electrode, which was found to be broken, was surgically removed by an inferior pericardiotomy [6].

Unlike the previously mentioned forms of extra-cardiac structure stimulation, our patient’s case occurred secondary to inadequate surgical handling of the original generator lead, which manifested as continuous stimulation of the rectus muscle, a rare electrophysiological complication. We hope that our experience will broaden our understanding of this rare complication and emphasize the need to carefully consider and pay close attention to all aspects of pacemaker implantation to avoid such technical errors.

## Conclusions

The mechanism behind the stimulation of the rectus muscle that occurred in our patient's case remains unclear. It could be either due to a lead insulation breach or mechanical irritation of the abdominal muscle by the abandoned lead that was attached to the contractile myocardium. Therefore, this explains the difficulty that was encountered in the prolonged process involving the diagnosis and treatment of our patient’s symptom, which persisted for over a decade. During the electrophysiology study, the only maneuver that successfully decreased the frequency of the rectus muscle stimulation was achieved by reducing the pacing rate. The site of the pacing, its polarity, or its amplitude were not contributing factors to the success of this maneuver.

Our case emphasizes the importance of adequate surgical handling of the abandoned epicardial lead by cutting the lead as short as possible, followed by capping the free tip. It also emphasizes that such complex cases involving electrophysiological complications necessitate a multidisciplinary approach involving cooperation between both the electrophysiology and cardiac surgery teams to formulate an appropriate plan of management to avoid unnecessary invasive interventions through proper preoperative evaluation. However, due to the lack of published literature on this subject, further studies and publications are necessary to help broaden our understanding of this dire yet rare technical complication, as early recognition of such technical errors that could arise during the replacement of permanent pacemakers is crucial.
